# *rac*-12-Selena-13,14-di­aza­tri­cyclo­[9.3.0.0^2,4^]tetra­deca-11,13-diene

**DOI:** 10.1107/S2414314620010810

**Published:** 2020-08-11

**Authors:** Heiner Detert, Dieter Schollmeyer

**Affiliations:** a Johannes Gutenberg University Mainz, Department of Chemistry, Duesbergweg 10-14, 55099 Mainz, Germany; University of Aberdeen, Scotland

**Keywords:** crystal structure, heterocycles, medium-sized ring, selenium

## Abstract

The centrosymmetric crystal structure of the title compound, C_11_H_16_N_2_Se, is built up from alternating strands of (*R,R*)- and (*S,S*)-enanti­omers. These strands, which propagate along the *c-*axis direction, are composed of homochiral mol­ecules related to each other by twofold screw axes. The shape of the mol­ecule is an almost planar unit around the selena­diazole ring with a hexa­methyl­ene chain as an arched handle.

## Abstract 2

The centrosymmetric crystal structure of the title compound, is built up from alternating strands of (*R,R*)- and (*S,S*)-enanti­omers. These strands, which propagate along the *c-*axis direction, are composed of homochiral mol­ecules related to each other by twofold screw axes. The shape of the mol­ecule is an almost planar unit around the selena­diazole ring with a hexa­methyl­ene chain as an arched handle.

## Graphical abstract

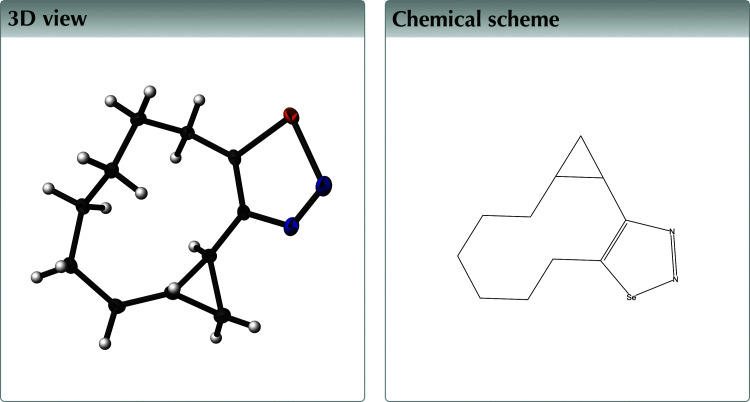

## Structure description

1,2,3-Selena­diazo­les are synthesized from SeO_2_-oxidation of semicarbazones (Yalpani *et al.*, 1971 ▸; Al-Smadi & Ratrout, 2004 ▸) and are important inter­mediates for the synthesis of medium-sized (Meier, 1972 ▸), heterocyclic (Detert, 2011 ▸), and strained cyclo­alkynes (Bissinger *et al.* 1988 ▸).

**Figure 1:**
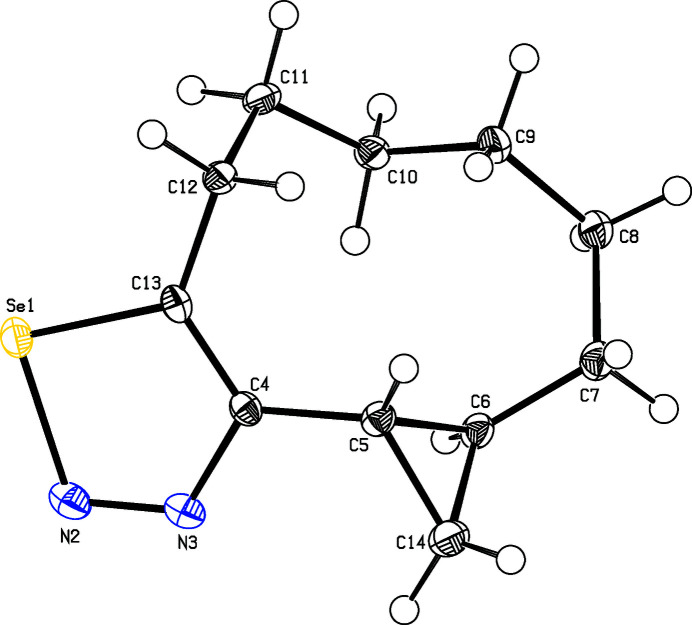
Perspective view of the title compound with displacement ellipsoids drawn at the 50% probability level.

The arbitrarily chosen asymmetric mol­ecule of the title compound (Fig. 1 ▸) has *S* configurations for atoms C5 and C6 but crystal symmetry generates a racemic mixture. The selena­diazole ring with the directly bound carbon atoms and one bond of the cyclo­propane ring is almost planar, with a maximum deviation from this plane of 0.037 (2) Å at C12 and the dihedral angle between the selena­diazole ring and the cyclo­propane ring is 69.0 (2)°. Though the carbocyclic part is of medium ring size, the hexa­methyl­ene tether appears to be free of Pitzer and Prelog strain.

**Figure 2:**
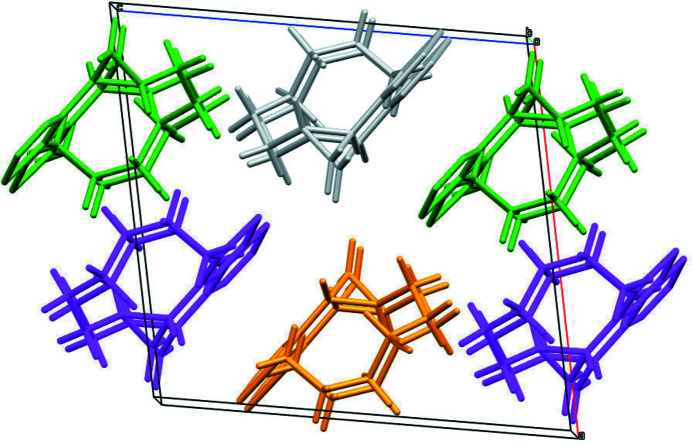
Packing diagram of the title compound viewed along the *b*-axis direction. Colour key: the asymmetric mol­ecule and its translation clones along [010] grey; mol­ecules generated by twofold screw axes green; mol­ecules generated by inversion symmetry orange; mol­ecules generated by *c*-glides purple.

No directional inter­actions beyond normal van der Waals contacts could be identified in the crystal. The packing consists of strands of homochiral mol­ecules, related to each other by twofold screw axes, propagating along the *c-*axis direction. The (*R,R*)- and (*S,S*)-enanti­omers alternate along the *a*-axis direction, being related by crystallographic *c*-glides (Fig. 2 ▸).

## Synthesis and crystallization

The title compound was prepared in ten steps from cyclo­octene according to Moore & Ward (1963 ▸), Moore & Bertelson (1962 ▸), Gardner & Narayana (1961 ▸), Detert & Meier (1997 ▸) and Whitham & Zaidlewicz (1972 ▸). Recrystallization from petroleum ether gave slightly pinkish crystals with m.p. 369 K.

## Refinement

Crystal data, data collection and structure refinement details are summarized in Table 1 ▸.

## Supplementary Material

Crystal structure: contains datablock(s) I, global. DOI: 10.1107/S2414314620010810/hb4354sup1.cif

Structure factors: contains datablock(s) I. DOI: 10.1107/S2414314620010810/hb4354Isup2.hkl

Supporting information file. DOI: 10.1107/S2414314620010810/hb4354Isup3.cml

CCDC reference: 2021398

Additional supporting information:  crystallographic information; 3D view; checkCIF report

## Figures and Tables

**Table 1:** Experimental details

Crystal data	
Chemical formula	C_11_H_16_N_2_Se
*M*_r_	255.22
Crystal system, space group	Monoclinic, *P*2_1_/*n*
Temperature (K)	120
*a*, *b*, *c* (Å)	12.0239 (6), 7.1317 (3), 12.6836 (7)
β (°)	101.104 (4)
*V* (Å^3^)	1067.27 (9)
*Z*	4
Radiation type	Mo *K*α
μ (mm^−1^)	3.48
Crystal size (mm)	0.55 × 0.47 × 0.10

Data collection	
Diffractometer	Stoe IPDS 2T
Absorption correction	Integration
*T*_min_, *T*_max_	0.228, 0.704
No. of measured, independent and observed [*I* > 2σ(*I*)] reflections	5541, 2570, 2364
*R*_int_	0.021
(sin θ/λ)_max_ (Å^−1^)	0.661

Refinement	
*R*[*F* ^2^ > 2σ(*F* ^2^)], *wR*(*F* ^2^), *S*	0.035, 0.097, 1.10
No. of reflections	2570
No. of parameters	127
H-atom treatment	H-atom parameters constrained
Δρ_max_, Δρ_min_ (e Å^−3^)	1.13, −0.90

Computer programs: *X-AREA* *WinXpose*, *Recipe* and *Integrate* (Stoe & Cie, 2019 ▸), *SIR2004* (Burla *et al.*, 2005 ▸), *SHELXL2018/3* (Sheldrick, 2015 ▸) and *PLATON* (Spek, 2020 ▸).

## full crystallographic data

### Crystal data

**Table:**

C_11_H_16_N_2_Se	*F*(000) = 520
*M**_r_* = 255.22	*D*_x_ = 1.588 Mg m^−^^3^
Monoclinic, *P*2_1_/*n*	Mo *K*α radiation, λ = 0.71073 Å
*a* = 12.0239 (6) Å	Cell parameters from 11220 reflections
*b* = 7.1317 (3) Å	θ = 2.9–28.5°
*c* = 12.6836 (7) Å	µ = 3.48 mm^−^^1^
β = 101.104 (4)°	*T* = 120 K
*V* = 1067.27 (9) Å^3^	Plate, colourless
*Z* = 4	0.55 × 0.47 × 0.10 mm

### Data collection

**Table:**

Stoe IPDS 2T diffractometer	2570 independent reflections
Radiation source: sealed X-ray tube, 12 x 0.4 mm long-fine focus	2364 reflections with *I* > 2σ(*I*)
Detector resolution: 6.67 pixels mm^-1^	*R*_int_ = 0.021
rotation method, ω scans	θ_max_ = 28.0°, θ_min_ = 3.3°
Absorption correction: integration	*h* = −15→15
*T*_min_ = 0.228, *T*_max_ = 0.704	*k* = −9→9
5541 measured reflections	*l* = −15→16

### Refinement

**Table:**

Refinement on *F*^2^	Primary atom site location: dual
Least-squares matrix: full	Hydrogen site location: inferred from neighbouring sites
*R*[*F*^2^ > 2σ(*F*^2^)] = 0.035	H-atom parameters constrained
*wR*(*F*^2^) = 0.097	*w* = 1/[σ^2^(*F*_o_^2^) + (0.0573*P*)^2^ + 1.4452*P*] where *P* = (*F*_o_^2^ + 2*F*_c_^2^)/3
*S* = 1.10	(Δ/σ)_max_ < 0.001
2570 reflections	Δρ_max_ = 1.13 e Å^−^^3^
127 parameters	Δρ_min_ = −0.90 e Å^−^^3^
0 restraints	

### Special details

**Table:**

Geometry. All esds (except the esd in the dihedral angle between two l.s. planes) are estimated using the full covariance matrix. The cell esds are taken into account individually in the estimation of esds in distances, angles and torsion angles; correlations between esds in cell parameters are only used when they are defined by crystal symmetry. An approximate (isotropic) treatment of cell esds is used for estimating esds involving l.s. planes.
Refinement. Hydrogen atoms attached to carbon atoms were placed at calculated positions and were refined in the riding-model approximation with C—H = 0.95 Å and *U*_iso_(H) = 1.2 *U*_eq_(C).

### Fractional atomic coordinates and isotropic or equivalent isotropic displacement parameters (Å^2^)

**Table:**

	*x*	*y*	*z*	*U*_iso_*/*U*_eq_	
Se1	0.01806 (2)	0.53889 (3)	0.19801 (2)	0.01717 (11)	
N2	0.08518 (19)	0.7735 (3)	0.24245 (18)	0.0195 (4)	
N3	0.16960 (17)	0.7494 (3)	0.31890 (17)	0.0167 (4)	
C4	0.1961 (2)	0.5686 (3)	0.35355 (19)	0.0131 (4)	
C5	0.2954 (2)	0.5360 (3)	0.4406 (2)	0.0144 (4)	
H5	0.337544	0.417298	0.433140	0.017*	
C6	0.2942 (2)	0.5921 (3)	0.55614 (18)	0.0149 (4)	
H6	0.225279	0.662016	0.567102	0.018*	
C7	0.3456 (2)	0.4626 (3)	0.6468 (2)	0.0177 (5)	
H7A	0.375621	0.539411	0.711012	0.021*	
H7B	0.410401	0.396039	0.626172	0.021*	
C8	0.2633 (2)	0.3164 (4)	0.67731 (19)	0.0178 (5)	
H8A	0.203057	0.382731	0.705866	0.021*	
H8B	0.305015	0.236704	0.735703	0.021*	
C9	0.2071 (2)	0.1890 (3)	0.58429 (19)	0.0173 (5)	
H9A	0.260533	0.170100	0.534790	0.021*	
H9B	0.192121	0.064881	0.613659	0.021*	
C10	0.09558 (19)	0.2684 (3)	0.52058 (18)	0.0154 (4)	
H10A	0.035621	0.252598	0.563508	0.018*	
H10B	0.105037	0.404482	0.509630	0.018*	
C11	0.0569 (2)	0.1748 (3)	0.41110 (19)	0.0170 (5)	
H11A	−0.022326	0.212211	0.382070	0.020*	
H11B	0.058059	0.037076	0.421121	0.020*	
C12	0.1307 (2)	0.2250 (3)	0.32802 (19)	0.0147 (4)	
H12A	0.210110	0.187617	0.356552	0.018*	
H12B	0.103796	0.153162	0.261179	0.018*	
C13	0.1270 (2)	0.4310 (3)	0.30231 (19)	0.0143 (4)	
C14	0.3690 (2)	0.6950 (4)	0.4927 (2)	0.0195 (5)	
H14A	0.451685	0.673076	0.511923	0.023*	
H14B	0.346890	0.824166	0.468684	0.023*	

### Atomic displacement parameters (Å^2^)

**Table:**

	*U*^11^	*U*^22^	*U*^33^	*U*^12^	*U*^13^	*U*^23^
Se1	0.01788 (15)	0.01843 (16)	0.01400 (15)	0.00192 (8)	0.00010 (10)	0.00198 (8)
N2	0.0245 (10)	0.0147 (9)	0.0200 (11)	0.0019 (8)	0.0059 (8)	0.0027 (8)
N3	0.0199 (10)	0.0135 (9)	0.0180 (10)	0.0013 (8)	0.0068 (8)	0.0017 (7)
C4	0.0150 (10)	0.0136 (9)	0.0112 (10)	0.0013 (8)	0.0038 (8)	0.0011 (8)
C5	0.0148 (10)	0.0156 (11)	0.0130 (11)	−0.0013 (8)	0.0034 (9)	−0.0015 (8)
C6	0.0164 (10)	0.0163 (10)	0.0117 (10)	−0.0020 (9)	0.0021 (8)	−0.0022 (8)
C7	0.0177 (11)	0.0213 (12)	0.0135 (11)	−0.0028 (9)	0.0017 (9)	−0.0002 (8)
C8	0.0199 (11)	0.0200 (11)	0.0130 (11)	−0.0020 (9)	0.0021 (9)	0.0013 (9)
C9	0.0218 (11)	0.0166 (10)	0.0128 (11)	−0.0008 (9)	0.0013 (9)	0.0013 (8)
C10	0.0170 (10)	0.0162 (10)	0.0136 (11)	−0.0025 (8)	0.0044 (9)	0.0010 (8)
C11	0.0184 (11)	0.0157 (10)	0.0166 (11)	−0.0058 (9)	0.0027 (9)	−0.0015 (9)
C12	0.0176 (10)	0.0127 (10)	0.0134 (10)	−0.0011 (8)	0.0022 (8)	−0.0013 (8)
C13	0.0154 (10)	0.0166 (10)	0.0109 (10)	0.0020 (9)	0.0027 (8)	0.0024 (8)
C14	0.0211 (11)	0.0197 (11)	0.0179 (12)	−0.0054 (10)	0.0040 (9)	−0.0016 (9)

### Geometric parameters (Å, º)

**Table:**

Se1—C13	1.841 (2)	C8—H8A	0.9900
Se1—N2	1.896 (2)	C8—H8B	0.9900
N2—N3	1.273 (3)	C9—C10	1.535 (3)
N3—C4	1.379 (3)	C9—H9A	0.9900
C4—C13	1.367 (3)	C9—H9B	0.9900
C4—C5	1.480 (3)	C10—C11	1.530 (3)
C5—C14	1.510 (3)	C10—H10A	0.9900
C5—C6	1.522 (3)	C10—H10B	0.9900
C5—H5	1.0000	C11—C12	1.544 (3)
C6—C14	1.507 (3)	C11—H11A	0.9900
C6—C7	1.511 (3)	C11—H11B	0.9900
C6—H6	1.0000	C12—C13	1.504 (3)
C7—C8	1.538 (3)	C12—H12A	0.9900
C7—H7A	0.9900	C12—H12B	0.9900
C7—H7B	0.9900	C14—H14A	0.9900
C8—C9	1.538 (3)	C14—H14B	0.9900
			
C13—Se1—N2	87.26 (10)	C8—C9—H9A	108.9
N3—N2—Se1	109.79 (16)	C10—C9—H9B	108.9
N2—N3—C4	118.1 (2)	C8—C9—H9B	108.9
C13—C4—N3	115.9 (2)	H9A—C9—H9B	107.7
C13—C4—C5	124.8 (2)	C11—C10—C9	113.5 (2)
N3—C4—C5	119.3 (2)	C11—C10—H10A	108.9
C4—C5—C14	121.9 (2)	C9—C10—H10A	108.9
C4—C5—C6	121.4 (2)	C11—C10—H10B	108.9
C14—C5—C6	59.64 (15)	C9—C10—H10B	108.9
C4—C5—H5	114.4	H10A—C10—H10B	107.7
C14—C5—H5	114.4	C10—C11—C12	113.84 (19)
C6—C5—H5	114.4	C10—C11—H11A	108.8
C14—C6—C7	120.4 (2)	C12—C11—H11A	108.8
C14—C6—C5	59.79 (15)	C10—C11—H11B	108.8
C7—C6—C5	119.6 (2)	C12—C11—H11B	108.8
C14—C6—H6	115.3	H11A—C11—H11B	107.7
C7—C6—H6	115.3	C13—C12—C11	112.17 (19)
C5—C6—H6	115.3	C13—C12—H12A	109.2
C6—C7—C8	114.7 (2)	C11—C12—H12A	109.2
C6—C7—H7A	108.6	C13—C12—H12B	109.2
C8—C7—H7A	108.6	C11—C12—H12B	109.2
C6—C7—H7B	108.6	H12A—C12—H12B	107.9
C8—C7—H7B	108.6	C4—C13—C12	127.5 (2)
H7A—C7—H7B	107.6	C4—C13—Se1	108.95 (17)
C7—C8—C9	114.6 (2)	C12—C13—Se1	123.47 (18)
C7—C8—H8A	108.6	C6—C14—C5	60.58 (15)
C9—C8—H8A	108.6	C6—C14—H14A	117.7
C7—C8—H8B	108.6	C5—C14—H14A	117.7
C9—C8—H8B	108.6	C6—C14—H14B	117.7
H8A—C8—H8B	107.6	C5—C14—H14B	117.7
C10—C9—C8	113.3 (2)	H14A—C14—H14B	114.8
C10—C9—H9A	108.9		
			
C13—Se1—N2—N3	0.23 (17)	C7—C8—C9—C10	−89.4 (3)
Se1—N2—N3—C4	0.2 (3)	C8—C9—C10—C11	163.53 (19)
N2—N3—C4—C13	−0.8 (3)	C9—C10—C11—C12	−70.9 (3)
N2—N3—C4—C5	178.6 (2)	C10—C11—C12—C13	−62.5 (3)
C13—C4—C5—C14	179.2 (2)	N3—C4—C13—C12	−175.5 (2)
N3—C4—C5—C14	−0.1 (3)	C5—C4—C13—C12	5.1 (4)
C13—C4—C5—C6	−109.3 (3)	N3—C4—C13—Se1	0.9 (3)
N3—C4—C5—C6	71.3 (3)	C5—C4—C13—Se1	−178.47 (18)
C4—C5—C6—C14	−111.1 (2)	C11—C12—C13—C4	89.1 (3)
C4—C5—C6—C7	138.9 (2)	C11—C12—C13—Se1	−86.8 (2)
C14—C5—C6—C7	−110.0 (2)	N2—Se1—C13—C4	−0.62 (17)
C14—C6—C7—C8	−159.1 (2)	N2—Se1—C13—C12	175.9 (2)
C5—C6—C7—C8	−88.8 (3)	C7—C6—C14—C5	108.7 (2)
C6—C7—C8—C9	56.9 (3)	C4—C5—C14—C6	110.3 (2)
